# Identification of molecular signatures associated with early relapse after complete resection of lung adenocarcinomas

**DOI:** 10.1038/s41598-021-89030-9

**Published:** 2021-05-05

**Authors:** Helen Pasternack, Christiane Kuempers, Mario Deng, Iris Watermann, Till Olchers, Mark Kuehnel, Danny Jonigk, Christian Kugler, Florian Stellmacher, Torsten Goldmann, Jutta Kirfel, Ole Ammerpohl, Sven Perner, Martin Reck

**Affiliations:** 1 Institute of Pathology, University Hospital Schleswig-Holstein, Campus Luebeck, Ratzeburger Allee 160, 23562 Luebeck, Germany; 2 LungenClinic Grosshansdorf, 22927 Grosshansdorf, Germany; 3 Airway Research Center North, Member of the German Center for Lung Research (DZL), 22927 Grosshansdorf, Germany; 4 Institute for Pathology, Hannover Medical School, 30625 Hannover, Germany; 5 Biomedical Research in Endstage and Obstructive Lung Disease Hannover (BREATH), Member of the German Center for Lung Research (DZL), 30625 Hannover, Germany; 6 Pathology, Research Center Borstel, Leibniz Lung Center, 23845 Borstel, Germany; 7 Institute of Human Genetics, Christian-Albrechts-University Kiel and University Medical Center Schleswig-Holstein, Campus Kiel, 24105 Kiel, Germany; 8 Present Address: Institute of Human Genetics, University Medical Center Ulm, 89073 Ulm, Germany

**Keywords:** Lung cancer, Lung cancer, Cancer genetics, Cancer epigenetics

## Abstract

The only potentially curative treatment for lung adenocarcinoma patients remains complete resection of early-stage tumors. However, many patients develop recurrence and die of their disease despite curative surgery. Underlying mechanisms leading to establishment of systemic disease after complete resection are mostly unknown. We therefore aimed at identifying molecular signatures of resected lung adenocarcinomas associated with the risk of an early relapse. The study comprised 89 patients with totally resected stage IA–IIIA lung adenocarcinomas. Patients suffering from an early relapse within two years after surgery were compared to patients without a relapse in two years. Patients were clinically and molecular pathologically characterized. Tumor tissues were immunohistochemically analyzed for the expression of Ki67, CD45, CD4, CD8, PD1, PD-L1, PD-L2 and CD34, by Nanostring nCounter PanCancer Immune Profiling Panel as well as a comprehensive methylome profiling using the Infinium MethylationEPIC BeadChip. We detected differential DNA methylation patterns as well as significantly differentially expressed genes associated with an early relapse after complete resection. Especially, CD1A was identified as a potential biomarker, whose reduced expression is associated with an early relapse. These findings might help to develop biomarkers improving risk assessment and patient selection for adjuvant therapy as well as establish novel targeted therapeutic strategies.

## Introduction

Lung cancer is the most common cancer-related cause of death worldwide. Non-small cell lung cancer (NSCLC) represents the predominant morphology with the most common histology of adenocarcinoma.

The only potentially curative treatment for lung adenocarcinoma patients remains complete resection of early-stage tumors. However, about half of the patients develop recurrence and die of their disease within five years despite curative surgery^1^. The risk for distant recurrence is even higher than that of local recurrence^2^. Underlying mechanisms that lead to establishment of systemic spread of disease after complete resection are mostly unknown and the current staging system fails to appropriately predict clinical outcome in individual patients^3^. Although, some biomarkers for post-surgical prognosis prediction were identified, none of them was transferred to routine clinical application yet^3–6^.

Adjuvant chemotherapy has shown to improve progression-free as well as overall survival of patients with stage IB, II and IIIA NSCLC^7–10^. However, appropriate molecular biomarkers for prediction of post-surgical outcome and selection of patients for adjuvant therapy are lacking. Furthermore, every surgery itself possesses a certain amount of risk and thus an accurate selection of those patients who are eligible for complete resection needs to be achieved^4^.

There seems to be a peak in post-surgical recurrence risk within the first year after resection, whereas other patients present with quite long relapse-free survival^2^. Therefore, we compared molecular features of cases with an early relapse ≤ 2 years after curative surgical resection with a group of control cases without a relapse within 2 years after surgery. We aimed at identifying biomarkers associated with a high risk of recurrence in order to improve individualized pre- and post-surgical patient management.

## Results

### Clinical characterization

98 tumor tissues of resected pulmonary adenocarcinomas were available. 49 of these patients developed early relapse (relapse ≤ 2 years after surgical resection). 40 patients did not develop relapse within 2 years after surgery (late relapse). For n = 9 patients, follow-up period was too short for classification in one of the two groups and the cases were therefore excluded from further analyses. Descriptive data of the 89 remaining patients including tumor diagnostics, typical confounders, overall survival and relapse-free survival are given in Table 1.

**Table 1:** Clinical characterization (TNM staging, confounder and survival).

Category	Early relapse (n = 49)	Late relapse (n = 40)
Stage IA	7	9
Stage IB	10	5
Stage IIA	14	11
Stage IIB	7	8
Stage IIIA	11	7
V0: without vascular invasion	31	23
V1: with vascular invasion	5	1
VX: vascular invasion not determined	13	16
L0: without lymphatic invasion	16	16
L1: with lymphatic invasion	20	8
LX: lymphatic invasion not determined	13	16
G1: well differentiated (Low grade)	2	1
G2: moderately differentiated (Intermediate grade)	6	13
G3: poorly differentiated (High grade)	40	25
G4: undifferentiated (High grade)	1	0
GX: G status not determined	0	1
Age [years]: average	64.0	65.3
Age [years]: standard deviation	10.2	7.9
Age [years]: median	63.9	65.2
Sex: male	37	18
Sex: female	12	22
Nicotine: yes	40	27
Nicotine: no	9	13
Packyears: average	39.5	40.3
Packyears: standard deviation	17.0	17.4
Packyears: median	40.0	40.0
Vital status: alive	21	37
Vital status: dead	28	3
Survival [months]: average	27.4	38.6
Survival [months]: standard deviation	15.6	9.7
Survival [months]: median	22.2	38.7
Time to relapse [months]: average	10.0	34.5
Time to relapse [months]: standard deviation	5.8	10.4
Time to relapse [months]: median	8.9	33.8

### Histological and immunohistochemical characterization

In the H&E stainings the different growth patterns lepidic, acinar, papillary, solid, micropapillary as well as combinations of them were seen. Due to the multitude of different pattern constellations and therefore small patient groups a correlation with the clinical outcome could not be drawn for this analysis.

Employing Ki-67 staining proliferation index of the tumors was determined. Density of vascularization was determined through CD34 analysis. Additionally, CD34 stainings were examined for organized vascularization patterns. Tumor infiltrating lymphocytes were identified using CD45 staining as well as further differentiated through CD4 and CD8 marking. Furthermore, it was documented for all three markers if positive cells were arranged diffuse or follicular and if the invasion front showed an emphasized staining. Detection of PD-L1 protein expression with two different antibodies (28-8 as well as SP142) showed consistent results. Additionally, expression of PD-L2 and PD1 was examined.

Correlation with the patients’ clinical outcome was examined for all immunohistochemically analyzed markers and revealed no association with an early relapse or a long relapse-free survival (data not shown).

### Molecular pathological characterization

FISH analyses revealed no rearrangement of *ALK*, *ROS1* and *RET* gene loci in any of the examined cases. Additionally, amplification of the *PDL1* gene was proven only in a single case (Table 2). Therefore, no data integration was performed for complete FISH analyses.

**Table 2:** Molecular pathological characterization (Sequencing and FISH analyses).

Category	Early relapse (n = 49)	Late relapse (n = 40)
*EGFR/KRAS* wildtype	25 (51%)	13 (33%)
*KRAS* exon 2 or 3 mutated (point mutations)	21 (43%)	17 (43%)
*EGFR* exon 18+20 mutated (point mutations)	0	1 (3%)
*EGFR* exon 19 mutated (deletions or duplication)	1 (2%)	5 (13%)
*EGFR* exon 20 mutated (point mutation or duplication)	0	2 (5%)
*EGFR* exon 21 mutated (point mutations)	2 (4%)	2 (5%)
*ALK/RET/ROS1* FISH wildtype	49 (100%)	40 (100%)
*ALK* rearrangement	0	0
*RET* rearrangement	0	0
*ROS1* rearrangement	0	0
*PD-L1* FISH wildtype	49 (100%)	39 (98%)
*PD-L1* amplification	0	1 (2%)

Mutational status of *EGFR* exon 18, 19, 20 and 21 as well as *KRAS* exon 2 and 3 were determined for all cases using Sanger sequencing of the corresponding gene sections. In total, fractions of 43% *KRAS* mutated and 15% *EGFR* mutated cases were present (Table 2). We observed a tendency for an association of a longer relapse-free survival with the presence of an *EGFR* mutation. Due to the respectively small case numbers per group this correlation could not be proven with regression models.

### DNA methylation analyses

**Figure 1:**
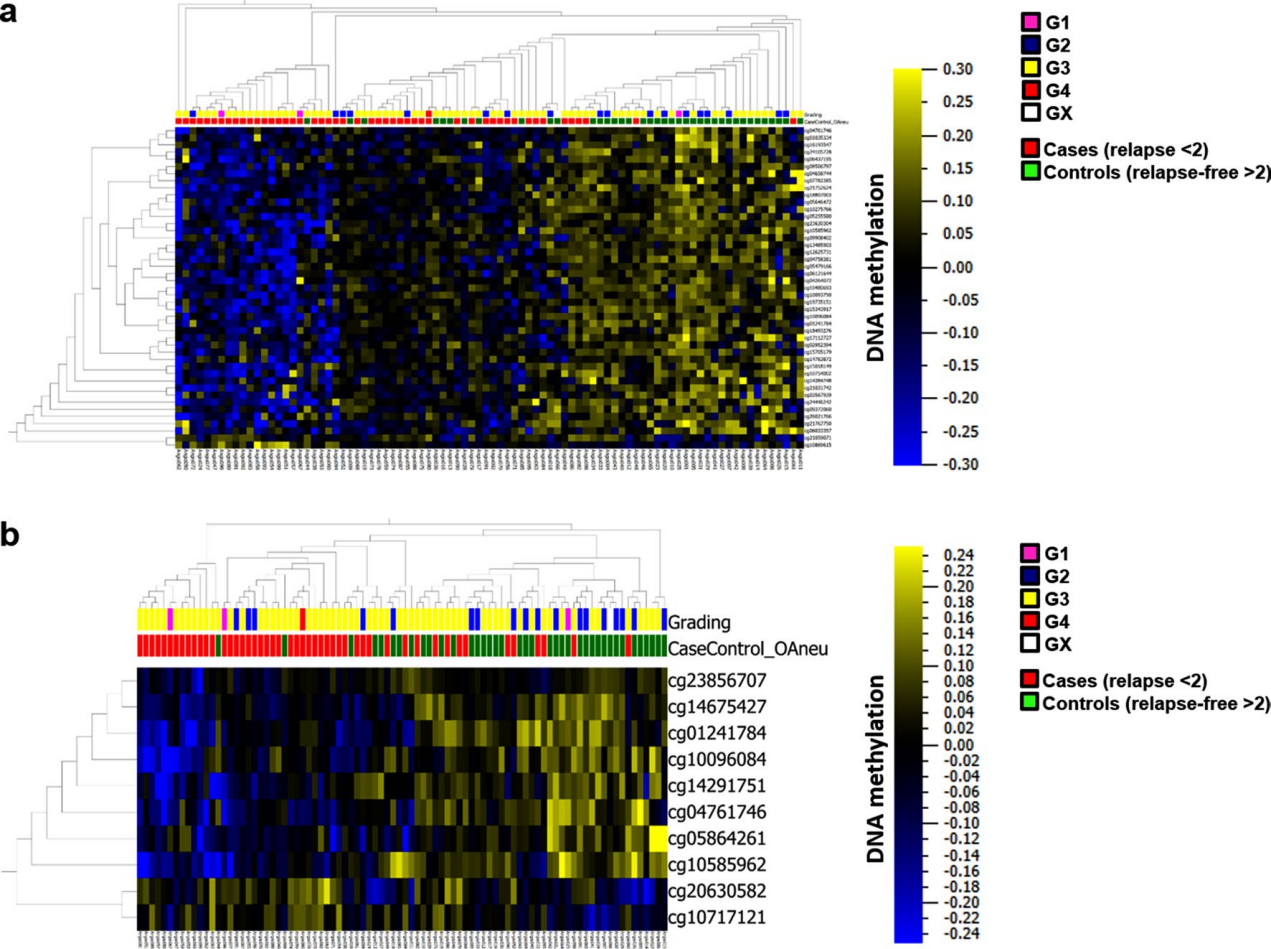
Methylome profiling (heatmaps: blue: low, yellow: high DNA methylation). (a) Hierarchical cluster analysis of DNA methylation data of 420 CpG loci differentially methylated between short-term (red boxes on top of heatmap) and long-term relapse-free survivors (green boxes). *p* < 4 × 10^−4^, σ/σ_max_>0.3; normalized presentation: mean = 0, scale: − 0.3–0.3. (**b**) Classification of short-term (red boxes on top of the heatmap) and long-term relapse-free survivors (green boxes) based on the DNA methylation pattern (random tree).

A differential methylation analysis (DMA) to identify loci differentially methylated in short-term and long-term relapse-free survivors identified 420 CpG loci (*p* < 4 × 10^−4^, σ/σ_max_ > 0.3) corresponding to 219 genes. 169 CpG loci were not allocated to a distinct gene (Supplementary Table S1). A hierarchical cluster analysis of methylation data based on these loci resulted essentially in three major branches (Fig. 1a), one preferentially containing cases with early relapses (96%), one carrying mainly late relapses (88%) and one mixed arm (71% early relapse), which argues for diverse DNA methylation patterns in the two groups of patients. A gene ontology analysis using the GOrilla-Tool^11,12^ indicated a significant enrichment of genes involved in signaling (GO:0023052, FDR < 7.34 × 10^−3^) in the set of 219 differentially methylated genes.

A subsequently performed string analysis (string-db.org) revealed numerous putative interactions between the 219 gene products (Supplementary Figure S1 and Supplementary Table S2). Putative interactions have been found in particular for components of signaling pathways (e.g. SHANK2, GRIK5, NRXN3).

Most aberrantly methylated genes were represented by a single CpG locus only. However, six CpG loci were located in CACNA1C, five in LDLRAD4, four in ADAMTS17, and three in COL23A1, NTRK2, PHACTR1, ANKS1B and APCDD1L-AS1 each (Supplementary Table S3).

In a second approach, three machine learning algorithms (support vector machine, SVM, K-nearest neighbor, KNN, and Random Tree, RDT) were applied to build a classifier for differentiating short-term from long-term relapse-free survivors (Fig. 1b). All classifiers sorted the samples into the particular categories; nevertheless 100% discrimination could not be reached. The best performing one of these algorithms (Random Tree) included ten CpG loci (cg20630582, cg23856707, cg01241784, cg10585962, cg14675427, cg10717121, cg14291751, cg10096084, cg04761746, cg05864261) and showed an accuracy of 0.71 in cases with an early relapse and 0.51 for cases with late relapse. In the epigenetic age of the tumors (difference between chronological age and DNAmAge) of long-term and short-term relapse-free survivors no significant differences were detected.

Information on the respective tumor grade was included in the heat maps (Fig. 1a,b) illustrating that clustering based on methylome profiling is not just reproducing tumor grades but provides additional value for individual risk stratification of patients.

### mRNA expression analyses

mRNA expression analyses using the nCounter PanCancer Immune Profiling Panel were performed for a total of 60 tumor samples (30 per patient cohort). The commercial panel was supplemented with the custom specific genes *FGFR1/2/3/4*, *PDGFRA/B*, *VEGFR1/2/3*, *KIT, RET*, *TGFBR1/2/3*, *BAMBI* and *VEGF-A*.

**Figure 2:**
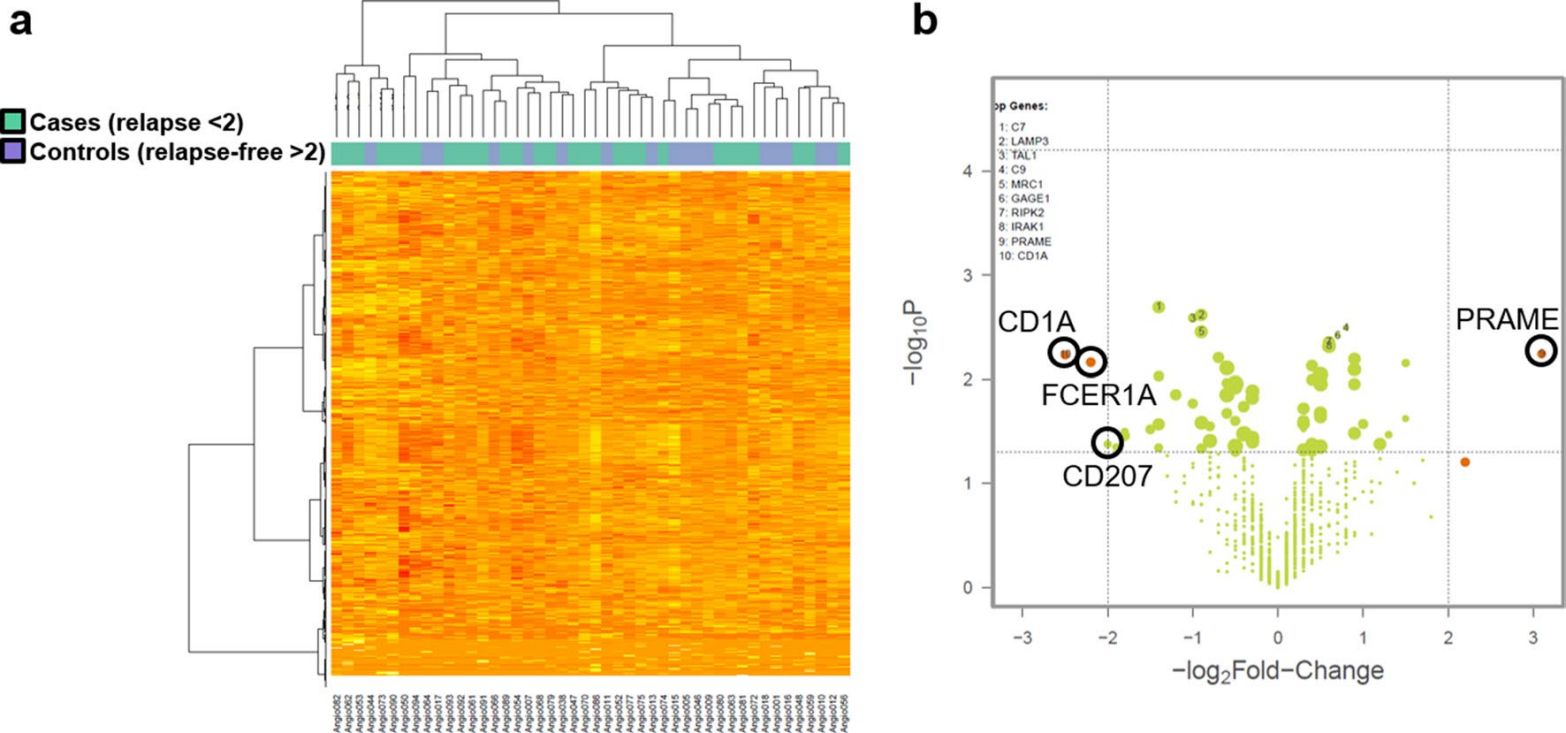
Immune profiling using the nCounter PanCancer Immune Profiling Panel. (**a**) Cluster analysis of normalized nanoString mRNA expression data between short-term (cases, light green boxes on top of heatmap) and long-term relapse-free survivors (controls, light blue boxes). (**b**) Vulcano plot of significantly differentially expressed candidate genes between the patient cohorts (*p* value <0.05 und foldchange >2).

Normalized nanoString mRNA expression data was analyzed in a cluster analysis. As shown in Fig. 2a no underlying global differential expression pattern between the two patient cohorts was identified. Even in random forest and regression analyses as well as after shifting the cut-off no classification into the two clinical groups based on the mRNA expression patterns could be achieved (data not shown).

**Figure 3:**
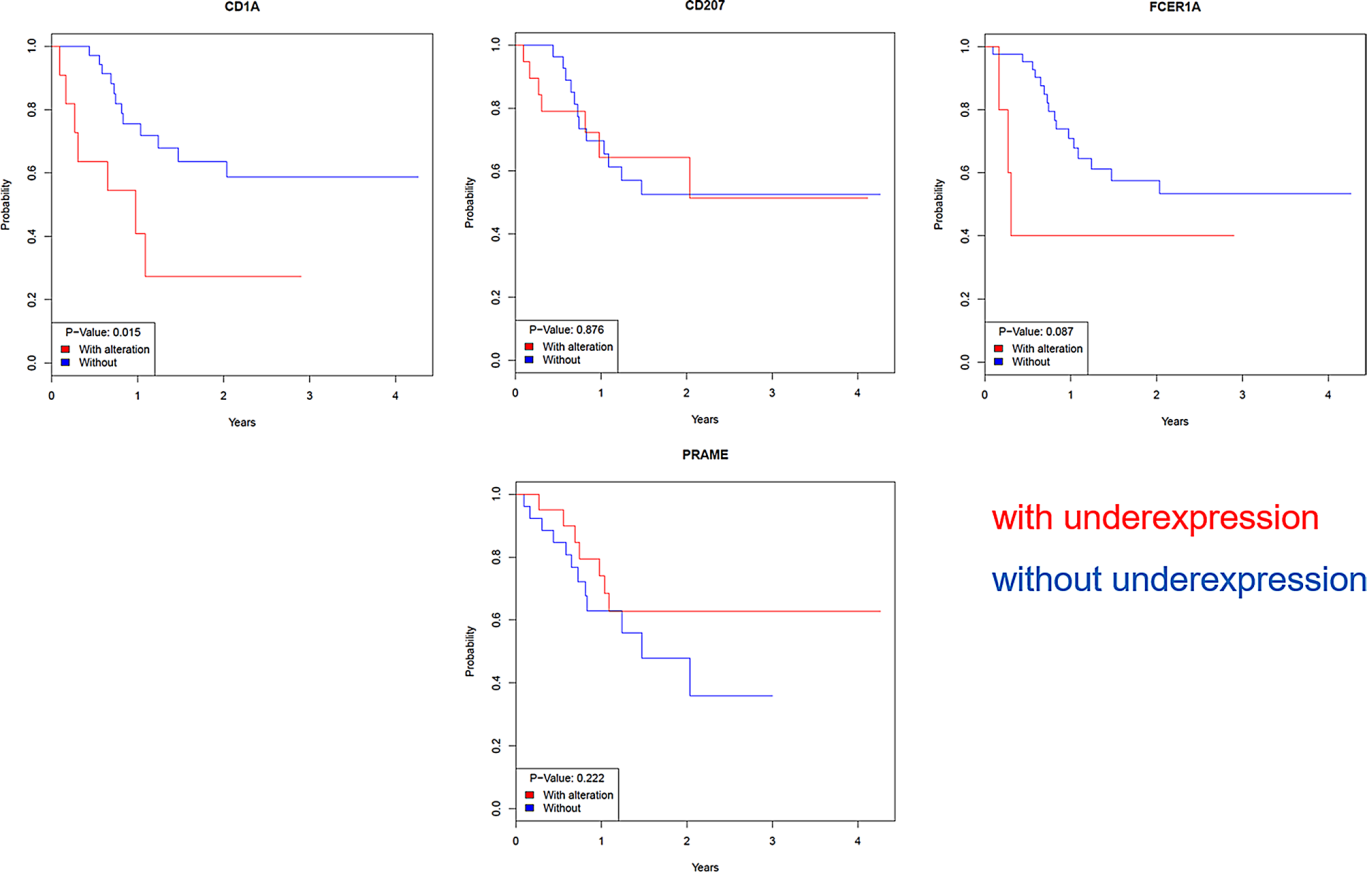
Correlation of mRNA expression of the four identified candidate genes *CD1A, CD207*, *FCER1A* and *PRAME* with clinical outcome of the patients (time to post-surgical relapse) using Kaplan-Meier estimator. Underexpression is defined as foldchange < −2 compared to mean expression of all reference genes.

Although no global expression pattern differentiating between the two patient cohorts could be seen, individual significantly differentially expressed candidate genes between the patient cohorts (*p* value < 0.05 und foldchange > 2) could be identified (Fig. 2b). According to that an early relapse within two years after R0 resection was associated with a low expression of *CD1A*, *CD207*, *FCER1A* and a high expression of *PRAME*. This differential expression is not accompanied with differential DNA methylation of corresponding gene loci. The respective correlation of mRNA expression of the four identified candidate genes with clinical outcome of the patients was examined using Kaplan–Meier estimator (Fig. 3). Especially for *CD1A* an association of low mRNA expression with a worse clinical outcome, meaning the occurrence of an early relapse, could be shown.

**Figure 4:**
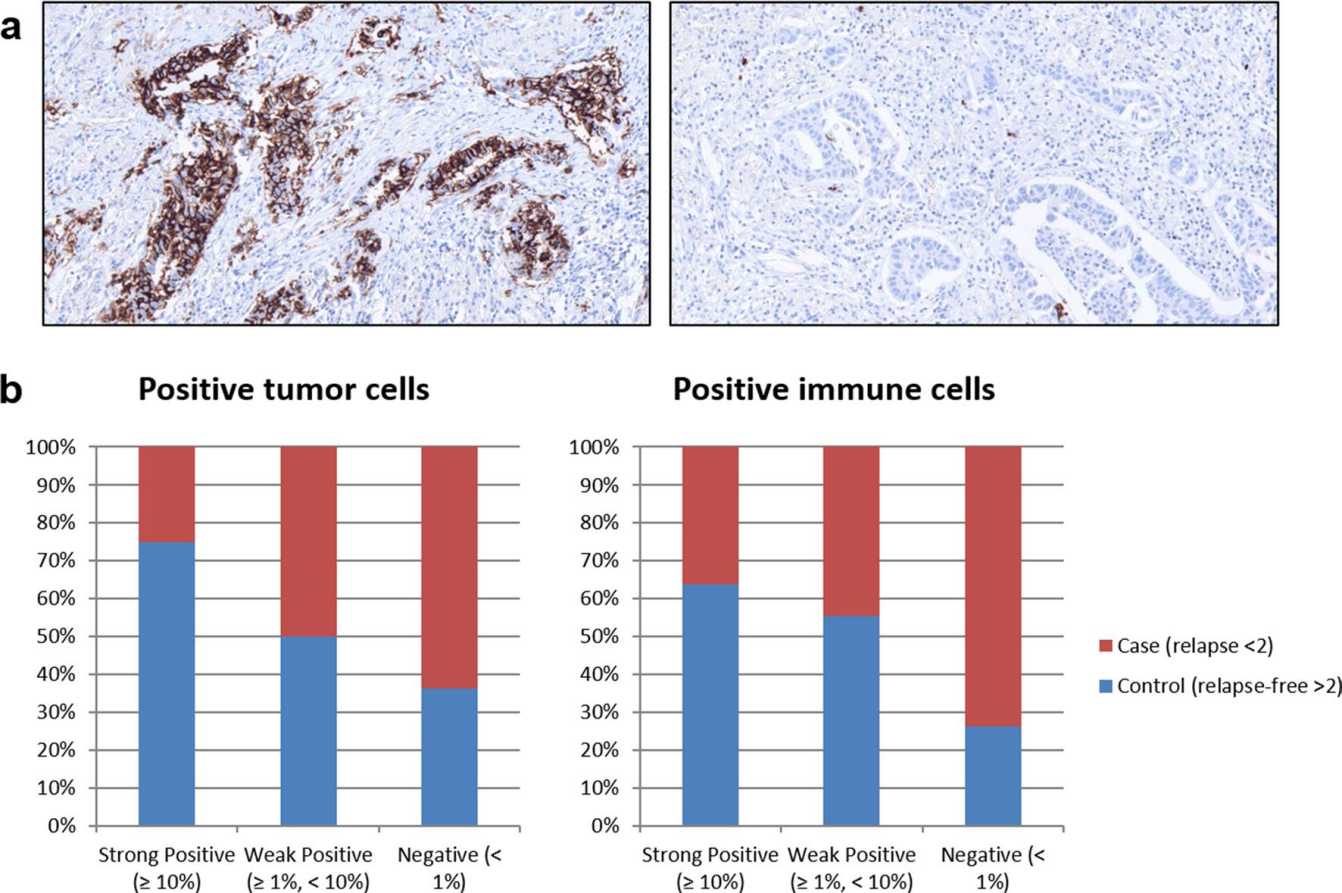
CD1A validation on protein level using immunohistochemical staining. (**a**) Representative CD1A expression patterns in a long-term relapse-free survivor with strong expression (left) and a short-term relapse-free survivor without expression (right). (**b**) CD1A expression was quantified as fraction of positive tumor cells as well as contribution of positive immune cells to the tumor area in short-term (cases, red) versus long-term relapse-free survivors (controls, blue).

The detected correlation between *CD1A* mRNA expression and clinical outcome was confirmed on protein level by immunohistochemistry. This validation revealed that an early relapse is much more frequent in CD1A negative than in positive cases (Fig. 4). Regression analysis using chi square test confirmed a highly significant correlation between loss of CD1A protein expression (< 1%) and occurrence of an early post-surgical relapse (*p* = 0.0226 for tumor cells and *p* = 0.0012 for immune cells). As the early relapse group is enriched for high grade and undifferentiated tumors (Table 1), we also performed chi-square test for correlation analysis between CD1A protein expression and tumor grading. This analysis revealed that immunohistochemical detection of CD1A expression in tumor as well as immune cells respectively is independent from grading as G1/G2 vs. G3/G4 tumor (*p* > 0.05).

## Discussion

A lot of patients suffer from early post-surgical recurrence after complete resection of early-stage lung adenocarcinomas, whereas other patients present with quite long relapse-free survival. The underlying molecular mechanisms differentiating these two patient cohorts are mostly unknown. Therefore, we compared molecular features of cases with an early relapse ≤ 2 years after curative surgical resection with a group of control cases without a relapse within 2 years after surgery.

In this study no association with an early relapse or a long relapse-free survival could be seen for any of the analyzed histological and immunohistochemical markers. This result is in line with another early biomarker study failing to show any significant association between survival and the expression of a biomarker panel, including angiogenesis markers^13^. In contrast, in a study on the prognostic relevance of angiogenesis in stage III NSCLC by Kreuter et al. microvessel density was a prognostic factor in the subgroup of R0-resected stage IIIA NSCLC patients^14^. In this small subgroup our data also indicate a tendency of less tumor vascularization in the early-relapse patient group. Also, the Ki-67 proliferation index was shown to predict the postoperative recurrence in early stage lung adenocarcinoma patients in previous studies^15,16^. This association is also visible in our data when comparing only stage I tumors. Although previous studies have demonstrated that the density of various immune cells in the tumor stromal compartment has significant prognostic relevance for NSCLC survival^17,18^, the herein presented data do not indicate a prognostic value of expression of immune checkpoint markers for post-surgical outcome of R0-resected lung adenocarcinoma patients.

In our cohort we detected fractions of 43% *KRAS* mutated and 15% *EGFR* mutated cases, although being slightly enriched for *KRAS* mutations, reflecting a typical distribution for a lung adenocarcinoma cohort^19^. We observed a tendency for an association of a longer relapse-free survival with the presence of an *EGFR* mutation. Due to the respectively small case numbers per group this correlation could not be proven with regression models. For stage III NSCLC with R0 resection it has already been shown that *EGFR* mutational status is more likely to be a predictive marker for response to treatment with tyrosine kinase inhibitors than a prognostic marker for post-surgical outcome^20^.

The differential methylation analysis (DMA) identified a set of 219 differentially methylated genes between the two patient groups. High Glutathione S-transferase P1 (GSTP1) DNA methylation was shown to be associated with a bad prognosis in NSCLC patients^21^. However, in the present study GSTP1 is not among the 219 differentially methylated genes. A string analysis revealed numerous putative interactions between the 219 gene products. These included proteins with a putative role in carcinogenesis, including lung cancer (e.g. CACNA1C^22^, FGFR2^23^, TLR9^24^ and CEACAMs^25^).

Among the differentially methylated genes CACNA1C was represented by six CpG loci, five loci were located in LDLRAD4, four in ADAMTS17, and three in COL23A1, NTRK2, PHACTR1, ANKS1B and APCDD1L-AS1 each. All of these genes have been reported playing a role in malignancies in humans, including lung cancer. CACNA1C encodes a calcium channel (calcium voltage-gated channel subunit alpha1 C). A copy number gain has been described in squamous cell carcinomas of the esophagus. Genetic alterations in this gene were also shown in lung adenocarcinoma indicating a putative function of CACNA1C in lung cancer^22^. The low-density lipoprotein receptor class A domain-containing protein 4 (LDLRAD4) has been described functioning as negative regulator of TGF-beta signaling by binding to SMAD2 and SMAD3 and therefore attenuating SMAD-recruitment to the TGF-beta receptor^26^. Liu et al. showed that LDLRAD4 also interacts with the ubiquitin ligase NEDD4 to affect TGF-beta signaling and promotes cell proliferation and migration^27^. Expression of ADAMTS17 has been found in fetal lung tissue as well as different adult normal tissues^28^. While Collagen XXIII expression has already been suggested as potential biomarker for NSCLC, APCDD1L-AS1 expression is of prognostic value in squamous cell carcinoma and NTRK2 has recently been reported as therapeutic target in combination with tyrosine kinase inhibitors in this tumor entity^29–31^. Also, for PHACTR1 and ANKS1B potential roles in lung cancer have been described before^32,33^. Interestingly, also two carcinoembryonic antigen genes (CEACAM6 and CEACAM7) showed differential DNA methylation patterns.

The best performing classifier for differentiating short-term from long-term relapse-free survivors used the Random Tree machine learning algorithm. It included ten CpG loci and showed an accuracy of 0.71 in cases with an early relapse and 0.51 for cases with late relapse. These results show the possibility to stratify the patients and samples according to their DNA methylation pattern and this stratification provides additional information to the existing grading system.

A disadvantage of this pilot study is the limited number of samples. Due to this fact, individual epigenetic alterations in single patients can appreciably affect the results. Furthermore, the applied algorithms required the classification of the donors into short-term and long-term relapse-free survivors. This classification was based on clinical experiences but not on biological or molecular criteria. Therefore, the classification was—to some extent—arbitrary. Consequently, individual differences in the constitution of the patients in the timespan until the relapse might have affected the outcome (in particular, if the 2-year limit has been barely reached or has been just missed).

A further validation of the DNA methylation-based outcome of this study, in particular in the context of supplementary gene expression data (either array or RNA-seq based) would be favorable to link the epigenetic to gene regulation and functional data.

mRNA expression analyses using the nCounter PanCancer Immune Profiling Panel revealed no underlying global differential expression pattern between the two patient cohorts. Nevertheless, individual significantly differentially expressed genes were identified. Especially for *CD1A* an association of low mRNA expression with a worse clinical outcome, meaning the occurrence of an early relapse within two years after R0 resection, could be shown. This association was also validated on protein level. CD1A protein expression in tumor as well as immune cells was independent from corresponding tumor grading but highly correlated with the occurrence of an early post-surgical relapse.

For the first time we could identify CD1A as a potential biomarker, whose reduced expression both on tumor and on tumor-infiltrating immune cells is associated with an early relapse after R0 resection of lung adenocarcinomas. CD1A is an MHC class I-like molecule capable of presenting lipid antigens and known to be expressed on antigen-presenting cells, e.g. dendritic cells. Lipid antigens were shown to be present in cancer cells and lipid-specific T cells play a role in anti-tumoral immune responses^34^. CD1A positive dendritic cell activity is regarded as one of the first steps in this immune response^35^. Additionally, the presence of a high number of antigen-presenting dendritic cells in the tumor was shown to be associated with increased disease-specific survival in resected non-small cell lung cancer^36^.

Given the fact that CD1A immunohistochemistry is already performed routinely in many institutes of pathology in order to detect CD1A expressing Langerhans cells, implementation of CD1A staining in routine diagnostics for resected early-stage lung adenocarcinoma is easily feasible. Therefore, loss of CD1A expression represents a novel potential biomarker to identify patients with an increased risk of post-surgical relapse that might benefit from adjuvant therapy. In addition to that CD1A expression might also be relevant in the context of immunotherapeutic approaches in lung cancer.

## Methods

### Study design and patient selection

The study was conducted at a single institution as retrospective, non-interventional case control study. It was conducted in accordance with the Declaration of Helsinki, and the protocol was approved by the ethics committee of the University Luebeck (14-043).

Initially, 235 patients with totally resected NSCLC adenocarcinomas stage I-IIIA (residual status: R0) between 2012 and 2014 were identified in the hospital information system of the LungenClinic Grosshansdorf. 179 patients signed the corresponding informed consent form allowing analysis of clinical data and tumor tissue. 100 of these patients were selected for follow-up. In total, 51 patients with early relapse ≤ 2 years after curative surgical resection have been identified (case group). In addition, 40 patients have been identified without a relapse within 2 years after curative surgical resection (control group). 9 patients were lost to follow-up. Patients were clinically and pathologically characterized (Table 1).

### Histological and immunohistochemical analyses

Histological as well as immunohistochemical analyses on formalin-fixed/paraffin-embedded (FFPE) tumor blocks were performed in the Pathologies of the University Medical Center Schleswig-Holstein, Campus Luebeck as well as Research Center Borstel, Leibniz Lung Center. Using H&E-stained slides the growth pattern of the analyzed tumors was determined. Lepidic, acinar, papillary, solid and micropapillary patterns were distinguished and were basis for grading: lepidic predominat = well differentiated (G1), acinar or papillary predominant = moderately differentiated (G2) and solid or micropapillary predominat = poorly differentiated (G3) according to the 2015 World Health Organization Classification of Lung Tumors. Grading was performed by a senior pathologist with great expertise in pulmonary pathology (FS). The expression of different markers on protein level was analyzed immunohistochemically using the Ventana Benchmark Ultra platform and OptiView HQ HRP method (Ventana Medical Systems, Tucson AZ, U.S.A.). Specific antibodies against CD34 (QBEnd/10, Ventana), Ki67 (30-9, Ventana), CD45 (2B11 & PD7/26, Ventana), CD4 (SP35, Ventana), CD8 (SP57, Ventana), PD-1 (NAT, Ventana), PD-L1 (SP142, Ventana as well as 28-8, Abcam, Cambridge UK), PD-L2 (ab200377, Abcam) and CD1A (EP3622, Ventana) were used.

Read-out for Ki-67 staining was the fraction of positive tumor cells. Using CD34 staining the number of vessels in 3 high power fields was determined. CD45 positive tumor infiltrating lymphocytes were quantified as fraction of the tumor area and by CD4 and CD8 marking the corresponding fraction of infiltrating lymphocytes were determined. Expression of the immune checkpoint markers PD-1, PD-L1 and PD-L2 was quantified as corresponding contribution of positive immune cells to the tumor area as well as the fraction of positive tumor cells (PD-L1 and PD-L2) or immune cells (PD1) respectively. CD1A protein expression was quantified as contribution of positive immune cells to the tumor area as well as fraction of positive tumor cells. To group the cases according to obtained expression values they were scored by arbitrary cut-offs as strong positive (≥ 10%), weak positive (≥ 1%, < 10%) or negative (< 1%).

### DNA and RNA extraction

For each case tissue areas with preferably high tumor cell content were selected for nucleic acid extractions using microdissection. Isolations of genomic DNA and mRNA were performed using the Maxwell RSC DNA FFPE resp. Maxwell RSC RNA FFPE Kits and the Maxwell RSC instrument (Promega, Fitchburg WI, U.S.A.). DNA samples were quantified with Nanodrop (ThermoFisher, Waltham MA, U.S.A.). Quantification of RNA samples was performed using the Qubit fluorimeter (ThermoFisher, Waltham MA, U.S.A.).

### Molecular pathological characterization

The possible presence of *ALK*, *RET* or *ROS1* translocations was investigated by fluorescence in-situ hybridization (FISH) using the corresponding ZytoLight SPEC Dual Color Break-Apart probes (*ALK* Z-2124, *RET* Z-2148, *ROS1* Z-2144, ZytoVision, Bremerhaven, Germany). Additionally, all samples were examined for amplifications of the *PDL1* gene locus using a custom-made FISH probe.

Mutations in the *EGFR* (exon 18, 19, 20, 21) and *KRAS* (Exon 2, 3) genes were detected with Sanger sequencing. Therefore, genomic DNA samples extracted from tumor tissues were amplified for relevant DNA sections of the *EGFR* and *KRAS* genes using specific primer pairs (*EGFR* 18: for 5′-TCCAGCATGGTGAGGGCTGAG, rev 5′-GGCTCCCCACCAGACCATG; *EGFR* 19: for 5′-GCTGGTAACATCCACCCAGA, rev 5′-GAGAAAAGGTGGGCCTGAG; *EGFR* 20: for 5′-CATGTGCCCCTCCTTCTG, rev 5′-GATCCTGGCTCCTTATCTCC; *EGFR* 21: for 5′-CCTCACAGCAGGGTCTTCTC, rev 5′-CCTGGTGTCAGGAAAATGCT; *KRAS* 2: for 5′-CCTTATGTGTGACATGTTC, rev 5′-TGGTCAGAGAAACCTTTATC; *KRAS* 3: for 5′-CCGTCATCTTTGGAGCAGGAAC, rev 5′-CTATAATTACTCCTTAATGTCAGC). Subsequently, Sanger sequencing reactions were performed employing the GenomeLab XP platform (AB Sciex, Framingham MA, U.S.A.).

### DNA methylation analyses

For stratification of the two clinical patient groups based on the DNA methylation pattern of tumor cells, a differential methylation analysis was performed by using the Infinium MethylationEPIC BeadChip (Illumina, San Diego CA, U.S.A.). Array hybridization was performed according to manufacturer's instruction; raw data collected by the iScan device was analyzed using Illumina's GenomeStudio software applying standard settings. Loci with detection.pvalue>0.01 as well as loci located on gonosomes were excluded from further analysis. Finally, data from 49 short-term relapse-free survivors as well as 39 long-term relapse-free survivors could be included into this study (608,811 loci each).

Further in-depth analyses were performed by using the Qlucore OMICS Explorer software (Qlucore, Lund, Sweden) as well as R- (www.r-project.org) and Perl-scripts (www.perl.org). CpG loci with p<4x10^−4^ and a relative variance (σ/σ_max_) >0.3 were considered differentially methylated. Machine learning algorithms (k-nearest neighbor, support vector machine and random tree) were applied to build classifiers for short- and long-term survivors. The GOrilla tool (http://cbl-gorilla.cs.technion.ac.il/) and STRING (www.string-db.org) were used for performing gene ontology analysis or interaction predictions, respectively (accessed in May 2019). The data set is available under GSE132690.

### mRNA expression analyses

mRNA expression analyses were performed using nCounter nanoString technology by means of the nCounter PanCancer Immune Profiling Panel (nanoString Technologies, Seattle WA, U.S.A.). The commercial panel was supplemented with the following anti-angiogenic target structures: *FGFR1/2/3/4*, *PDGFRA/B*, *VEGFR1/2/3*, *KIT*, *RET*, *TGFBR1/2/3*, *BAMBI*, *VEGFA*.

Due to in some cases low RNA concentrations 60 tumor samples could be examined by nanoString technology (RNA input: 45 ng). From both patient cohorts 30 patients were analyzed each. Readout of the cartridges was performed in the Institute for Pathology, Hannover Medical School. Obtained expression data has been normalized using the NanoStringNorm package for the R programming language with default parameters, following the suggested protocol. Code counts have been normalized by geometric mean, while background has been reduced with 2 standard deviations. From the normalized nanoString mRNA data genes with differential expression between the two patient cohorts were identified (p value <0.05 and foldchange >2). Cluster analysis as well as random forest and regression analyses were performed in order to detect global differential expression patterns. A correlation of the mRNA expression of identified differentially expressed candidate genes with the clinical outcome was examined using Kaplan-Meier estimator.

## Supplementary information

Supplementary information .

Supplementary Figure 1.

Supplementary Table 1.

Supplementary Table 2 .

Supplementary Table 3.

## Data Availability

Upon publication the methylome data set generated and analyzed during the current study will be openly available in Gene Expression Omnibus (GEO) at https://www.ncbi.nlm.nih.gov/geo/, reference number GSE132690. Other datasets generated and analyzed during the current study are available from the corresponding author on reasonable request.
