# Supplementary material for: Identification of molecular signatures associated with early relapse after complete resection of lung adenocarcinomas
Source: Sci Rep. 2021 May 5;11:9532. doi: 10.1038/s41598-021-89030-9 (PMC8099905; doi:10.1038/s41598-021-89030-9)
Supplement: Supplementary file 1 — Supplementary information . [file 41598_2021_89030_MOESM1_ESM.docx]

**Supplementary Information**

Supplementary Figure S1: String analysis of differentially methylated genes.

Supplementary Table S1: List of differentially methylated CpG loci.

Supplementary Table S2: Analysis results of db-STRING.

Supplementary Table S3: Number of affected CpG loci in genes differentially methylated.
