# Supplementary figures and images for: Identification of molecular signatures associated with early relapse after complete resection of lung adenocarcinomas
Source: Sci Rep. 2021 May 5;11:9532. doi: 10.1038/s41598-021-89030-9 (PMC8099905; doi:10.1038/s41598-021-89030-9)

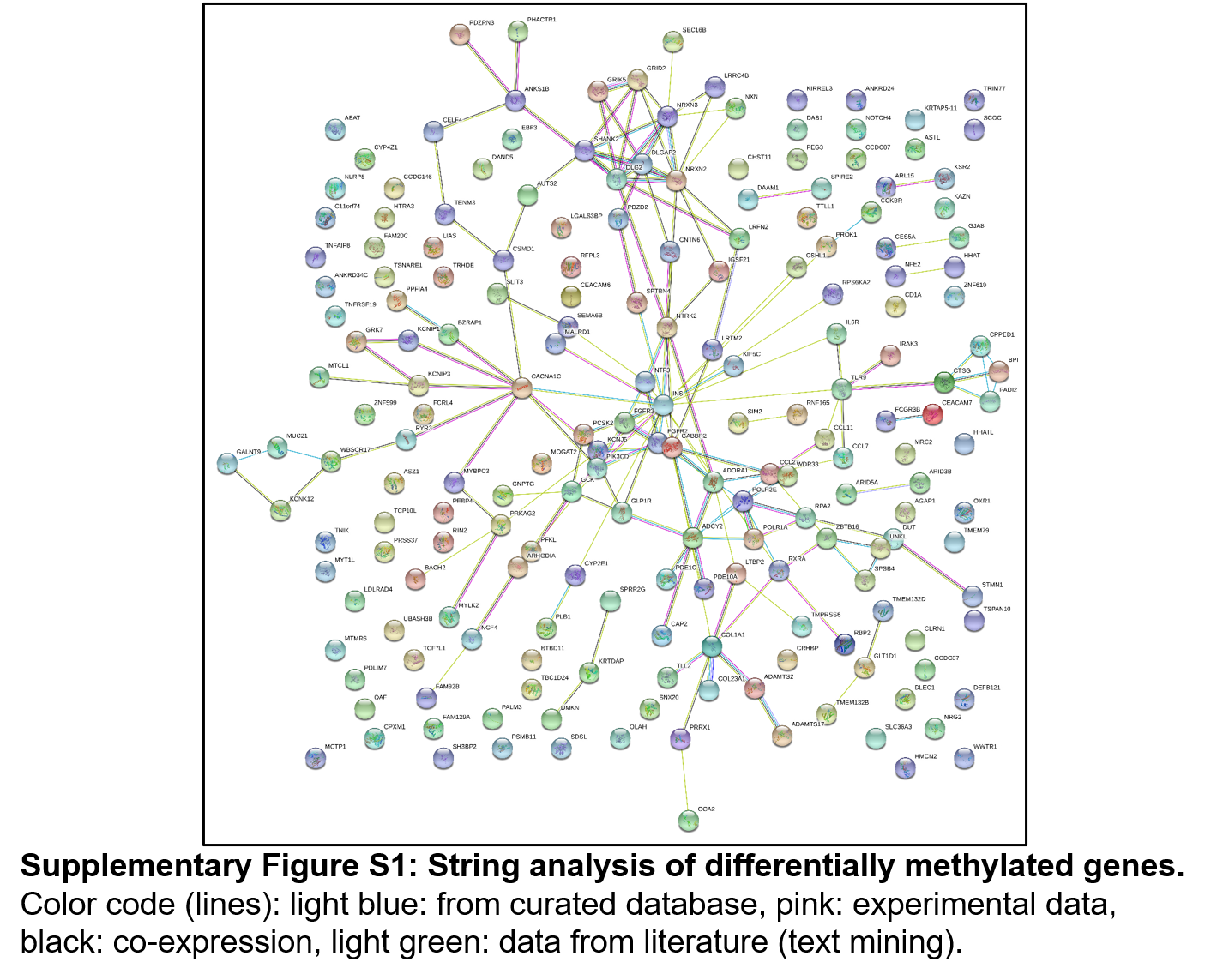

Supplement: Supplementary file 2 — Supplementary Figure 1. [file 41598_2021_89030_MOESM2_ESM.tif]
